# Polymeric Nanoparticles with ROS‐Responsive Prodrug and Platinum Nanozyme for Enhanced Chemophotodynamic Therapy of Colon Cancer

**DOI:** 10.1002/advs.202001853

**Published:** 2020-09-06

**Authors:** Ying Hao, Yuwen Chen, Xinlong He, Yongyang Yu, Ruxia Han, Yang Li, Chengli Yang, Danrong Hu, Zhiyong Qian

**Affiliations:** ^1^ State Key Laboratory of Biotherapy and Cancer Center West China Hospital Sichuan University, and Collaborative Innovation Center of Biotherapy Chengdu 610041 P. R. China; ^2^ Department of Gastrointestinal Surgery West China Hospital Sichuan University Chengdu 610041 P. R. China

**Keywords:** chemophotodynamic therapy, colon cancer, platinum nanozymes, polymeric nanoparticles, ROS‐responsive prodrugs

## Abstract

The combination of chemotherapy and photodynamic therapy (PDT) has promising potential in the synergistic treatment of cancer. However, chemotherapy and photodynamic synergistic therapy are impeded by uncontrolled chemotherapeutics release behavior, targeting deficiencies, and hypoxia‐associated poor PDT efficacy in solid tumors. Here, a platinum nanozyme (PtNP) loaded reactive oxygen species (ROS)‐responsive prodrug nanoparticle (CPT‐TK‐HPPH/Pt NP) is created to overcome these limitations. The ROS‐responsive prodrug consists of a thioketal bond linked with camptothecin (CPT) and photosensitizer‐2‐(1‐hexyloxyethyl)‐2‐devinyl pyropheophorbide‐a (HPPH). The PtNP in CPT‐TK‐HPPH/Pt NP can efficiently catalyze the decomposition of hydrogen peroxide (H_2_O_2_) into oxygen to relieve hypoxia. The production of oxygen can satisfy the consumption of HPPH under 660 nm laser irradiation to attain the on‐demand release of CPT and ensure enhanced photodynamic therapy. As a tumor diagnosis agent, the results of photoacoustic imaging and fluorescence imaging for CPT‐TK‐HPPH/Pt NP exhibit desirable long circulation and enhanced in vivo targeting. CPT‐TK‐HPPH/Pt NPs effectively inhibit tumor proliferation and growth in vitro and in vivo. CPT‐TK‐HPPH/Pt NP, with its excellent ROS‐responsive drug release behavior and enhanced PDT efficiency can serve as a new cancer theranostic agent, and will further promote the research of chemophotodynamic synergistic cancer therapy.

## Graphical abstract

The platinum nanozyme (PtNP) loaded reactive oxygen species‐responsive prodrug nanoparticle (CPT‐TK‐HPPH/Pt NP) which could achieve the “on‐demand” release of CPT and relieve hypoxia to enhance photodynamic therapy efficiency, exhibits desirable long circulation and effectively inhibits tumor proliferation and growth in vitro and in vivo, has great promising potential in chemophotodynamic synergistic cancer therapy.

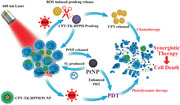

## 1 Introduction

Accounting for 10% of all cancer cases worldwide, colon cancer has become the third most common cancer with high mortality and morbidity. Chemotherapy is one of the common ways to treat colon cancer.^[^ ^1^ ^]^ However, the use of chemotherapeutics, such as irinotecan and topotecan derivatives, which are camptothecin (CPT) analogs, is disadvantageous because of uncontrolled release behavior and poor targeting ability, which easily induce systemic toxicity, immunosuppression, and drug resistance.^[^ ^2^ ^]^ To date, numerous studies have confirmed that combined therapy holds promising prospects in cancer treatment.^[^ ^3^ ^]^ Notably, photodynamic therapy (PDT), a non‐invasive method for cancer therapy, can generate reactive oxygen radicals (ROS) under light irradiation, which causes irreversible damage to tumor cells.^[^ ^4^ ^]^ However, the hypoxia feature of solid tumors is a key obstacle to PDT efficiency.^[^ ^5^ ^]^ Therefore, developing a chemophotodynamic synergistic therapy system that can ensure the on‐demand release of chemotherapeutics and enhanced ROS generation for photodynamic therapy is urgent and essential.

Interestingly, prodrugs can release bioactive drugs on‐demand at specific sites to reduce systemic toxicity, this is done by using the special properties of the tumor microenvironment, such as pH value, glutathione concentration, and specific overexpressed enzymes, or exogenous stimulation, such as light, heat, and ultrasound.^[^ ^6^ ^]^ Contemporarily, red or near‐infrared light, with the advantages of high tissue permeability, minimal phototoxicity, and the indirect destruction of chemical bonds, is an attractive driving force for controlling the release behavior of drugs.^[^ ^7^ ^]^ In addition, under laser irradiation, photosensitizers can generate ROS and effectively decompose ROS‐responsive covalent bonds, such as thioketal, arylboron ester, thioether, diselenide ether, and peroxyacetate.^[^ ^8^ ^]^ We achieved this by introducing an ROS‐responsive prodrug (CPT‐TK‐2‐(1‐hexyloxyethyl)‐2‐devinyl pyropheophorbide‐a (HPPH)) that consisted of thioketal bond linked CPT and photosensitizer HPPH. The HPPH was a potential photosensitizer with good photodynamic activity, a high penetration rate for tumor tissue, and low phototoxicity.^[^ ^9^ ^]^ Specifically, the chemotherapeutics CPT is a topoisomerase I inhibitor,^[^ ^10^ ^]^ which stabilized the topoisomerase I‐DNA complex and prevented DNA replication and RNA synthesis, thereby killing tumor cells. CPT is also a hypoxia inducible factor‐1a (HIF‐1a) inhibitor,^[^ ^11^ ^]^ which may enhance the cytotoxicity of HPPH. In addition, hypoxia is the main factors that affects the efficiency of photodynamic therapy. Some strategies, such as generating oxygen in situ or delivering exogenous oxygen, have been developed to ameliorate tumor hypoxia.^[^ ^12^ ^]^ However, factors such as the poor stability or rapid consumption of oxygen generator materials, oxygen leakage, and low efficiency in the production of exogenous oxygen still dampen the efficacy of PDT.^[^ ^13^ ^]^ The small platinum nanozyme (PtNP) has been extensively investigated owing to its inherent advantages of efficient catalytic activity and high stability, which may have great potential in PDT therapy.^[^ ^14^ ^]^

**Figure 1:**
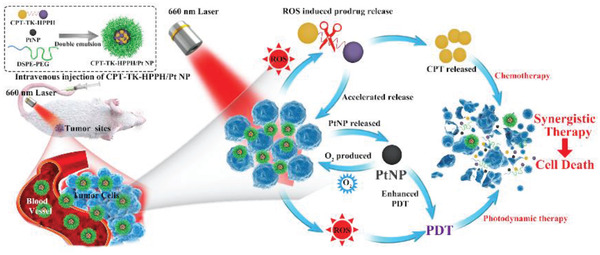
Schematic illustration of colon cancer treatment using CPT‐TK‐HPPH/Pt NP under 660 nm laser irradiation for 5 min.

In this study, we created a PtNP loaded ROS‐responsive prodrug nanoparticle (CPT‐TK‐HPPH/Pt NP) to attain enhanced chemophotodynamic therapy for colon cancer. As shown in **Figure** **1**, under 660 nm laser irradiation, the prodrug produced ROS that was effective for enhancing photodynamic therapy and controlled the release behavior of the CPT in the prodrug for attaining improved chemophotodynamic therapy for colon cancer. Importantly, the PtNP in the CPT‐TK‐HPPH/Pt NP catalyzed the decomposition of hydrogen peroxide (H_2_O_2_) to obtain oxygen for relieving hypoxia in tumor tissue and enhanced PDT efficiency. The production of oxygen satisfied the consumption of HPPH under 660 nm laser irradiation to ensure on‐demand release of CPT and enhanced photodynamic therapy. The designed CPT‐TK‐HPPH/Pt NP, with its excellent ROS‐responsive drug release behavior and enhanced PDT efficiency, can serves as a new strategy for the clinical treatment of colon cancer.

## 2 Results and Discussion

### 2.1 Synthesis and Characterization of the Prodrug

We synthesized the ROS‐responsive prodrug CPT‐TK‐HPPH and the non‐responsive prodrug CPT‐CC‐HPPH using 3,3′‐[(1‐methylethylidene)bis(thio)]bis‐propanoic acid and 1,6‐hexanediol, respectively. The ^1^H NMR spectra exhibited in Figure S3 (Supporting Information) demonstrated that CPT‐TK‐HPPH and CPT‐CC‐HPPH were successfully obtained.

### 2.2 Preparation and Characterization of Platinum Nanozyme

To improve photodynamic therapeutic efficiency, small PtNP, which were revealed to have good catalytic activity, were synthesized with sodium borohydride using a reduction method.^[^ ^14c^ ^]^ As shown in Figure S4A (Supporting Information), the hydrodynamic diameter of the PtNP measured using dynamic light scattering (DLS) was ≈14 nm with a narrow polydispersity index (PDI). As shown in transmission electron microscopy (TEM) imaging, the PtNP exhibited a uniform morphology with a size of 4 nm (Figure S4B, Supporting Information), in addition, crystalline fringes with interplanar spacings were observed in high‐resolution TEM imaging, as seen in Figure S4C (Supporting Information). Additionally, we evaluated the in vitro cell viability of PtNP on mouse fibroblast cell line 3T3 cells, shown in Figure S4D (Supporting Information), and the results demonstrated that the cell viability was higher than 70% even when the PtNP concentration was 40 µg mL^−1^ and after incubation for 48 h, which implied that the as‐prepared PtNP was safe for 3T3 cells. We also measured the absorbance of the mixture (tetramethylbenzidine (TMB), H_2_O_2_, and PtNP, at different concentration) at 652 nm to investigate the oxygen production ability of PtNP. When the PtNP decomposed the H_2_O_2_ and producing oxygen, the oxygen catalyzed the TMB solution and induced absorbance at 652 nm. The absorbance change at 652 nm was recorded at different times, as shown in Figure S4E (Supporting Information). The results exhibited that the catalytic capacity of PtNP was concentration‐dependent, and the PtNP still maintained catalytic activity at a concentration of 3 ng mL^−1^, which further showed that PtNP has great potential for producing oxygen in tumor tissue, thereby enhancing the efficiency of photodynamic therapy.

### 2.3 Preparation and Characterization of Polymeric Nanoparticle

**Figure 2:**
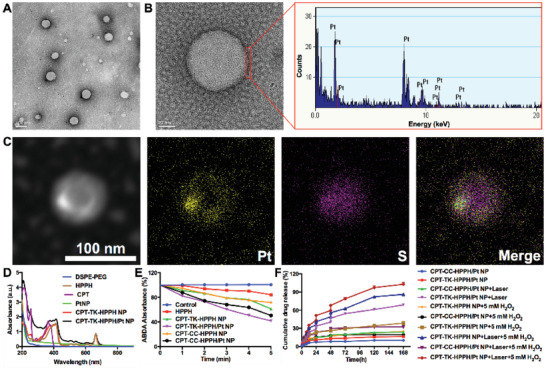
Characterization of CPT‐TK‐HPPH/Pt NP. A) TEM image (scale bar: 100 nm), B) magnified TEM image (scale bar: 20 nm) and energy dispersive X‐ray spectroscopy of the local area of a CPT‐TK‐HPPH/Pt NP, C) dark‐field TEM image and corresponding TEM elemental mappings of the Pt and S signals of CPT‐TK‐HPPH/Pt NP (scale bar: 100 nm). D) UV–vis absorbance spectra of DSPE‐PEG, HPPH, CPT, PtNP, CPT‐TK‐HPPH NP, and CPT‐TK‐HPPH/Pt NP, E) ABDA absorbance of control, HPPH, CPT‐TK‐HPPH NP, CPT‐TK‐HPPH/Pt NP, CPT‐CC‐HPPH NP, and CPT‐CC‐HPPH/Pt NP groups, F) release behavior of CPT‐TK‐HPPH NP, CPT‐TK‐HPPH/Pt NP, and CPT‐CC‐HPPH/Pt NP under different conditions. Data in (D) and (E) are presented as mean ± SD (*n* = 3).

The ROS‐responsive prodrug CPT‐TK‐HPPH and PtNP were loaded into amphiphilic polymer distearylphosphatidylethanolamine–polyethylene glycol (DSPE‐PEG), which possesses the characteristics of prolonging blood circulation time and tumor residence time, producing the polymeric nanoparticle CPT‐TK‐HPPH/Pt NP. The hydrodynamic diameter and zeta potential of CPT‐TK‐HPPH/Pt NP were 179 nm (PDI = 0.207) and −40 mV, respectively. The TEM imaging, shown in **Figure** **2**A, revealed that the CPT‐TK‐HPPH/Pt NP had a uniform size of ≈100 nm, and we analyzed the energy dispersive X‐ray spectroscopy of the local area of a CPT‐TK‐HPPH/Pt NP (Figure 2B), the results demonstrated that the PtNP were coated in the outer layer of nanoparticles as designed. Meanwhile, the TEM elemental mappings (Figure 2C) indicated that the prodrug CPT‐TK‐HPPH (S element) and PtNP (Pt elements) were successfully loaded into the nanoparticle. We further prepared a CPT‐TK‐HPPH loaded nanoparticle (CPT‐TK‐HPPH NP), a CPT‐CC‐HPPH loaded nanoparticle (CPT‐CC‐HPPH NP), and a CPT‐CC‐HPPH and PtNP co‐loaded nanoparticle (CPT‐CC‐HPPH/Pt NP) as the control. The particle size and zeta potential were shown in Figure S5 (Supporting Information). In addition, the drug loading capacity and encapsulation efficiency of the polymeric nanoparticles were summarized in **Table** **1**. The drug loading capacities of PtNP and the prodrug in the CPT‐TK‐HPPH/Pt NP were 0.744% and 9.92%, with the encapsulation efficiency of 99.2% and 99.2%, respectively. We also recorded the ultraviolet (UV) absorption spectra of DSPE–PEG, HPPH, CPT, PtNP, CPT‐TK‐HPPH NP, and CPT‐TK‐HPPH/Pt NP, as shown in Figure 2D. HPPH, CPT‐TK‐HPPH NP, and CPT‐TK‐HPPH/Pt NP had characteristic peaks at 660 nm, which could enhance photodynamic therapy under 660 nm laser irradiation.

**Table 1:** Characterization of nanoparticles

				PtNP		Prodrug	
Samples	DLS^a)^ [nm]	PDI^b)^	Zeta potential [mV]	DL [%]^c)^	EE [%]^d)^	DL [%]^e)^	EE [%]^f)^
CPT‐CC‐HPPH NP	119.23 ± 1.63	0.183 ± 0.013	−43.70 ± 0.62	/	/	9.94	99.4
CPT‐CC‐HPPH/Pt NP	193.83 ± 0.76	0.207 ± 0.013	−42.00 ± 0.85	0.744	99.2	9.93	99.3
CPT‐TK‐HPPH NP	156.03 ± 0.55	0.132 ± 0.006	−48.57 ± 1.17	/	/	9.93	99.3
CPT‐TK‐HPPH/Pt NP	179.67 ± 2.45	0.207 ± 0.007	−40.63 ± 0.65	0.744	99.2	9.92	99.2

^a)^ The particle size of the nanoparticle determined by dynamic light scattering;

^b)^ The polydispersity index (PDI) of particle size;

^c)^ The drug loading capacity of PtNP;

^d)^ The encapsulation efficiency of PtNP;

^e)^ The drug loading capacity of prodrug;

^f)^ The encapsulation efficiency of prodrug.

### 2.4 ROS Generation In Vitro

The production of the ROS directly affected the photodynamic therapy efficiency. 9,10‐Anthracenediyl‐bis(methylene)‐dimalonic acid (ABDA) was applied to measure the ROS generation ability in vitro, as ABDA could be oxidized by ROS, which would decrease the absorption peak at 378 nm.^[^ ^15^ ^]^ Accordingly, we used ABDA to investigate the ROS generation ability of HPPH, CPT‐TK‐HPPH NP, CPT‐TK‐HPPH/Pt NP, CPT‐CC‐HPPH NP, and CPT‐CC‐HPPH/Pt NP under 660 nm laser irradiation with a density of 200 mW cm^−2^. The UV–vis absorption spectra are presented in Figure S6 (Supporting Information). We further calculated the change in UV–vis absorbance at 378 nm. The results, shown in Figure 2E, revealed that the absorbance of ABDA at 378 nm under laser irradiation did not considerably change, which demonstrated that the ABDA did not affect the ROS generation ability. When the aforementioned components were irradiated under a 660 nm laser for 5 min, the absorbance of HPPH, CPT‐CC‐HPPH NP, CPT‐CC‐HPPH/Pt NP, CPT‐TK‐HPPH NP, and CPT‐TK‐HPPH/Pt NP at 378 nm decreased to 84%, 72%, 51%, 62%, and 42%, respectively, which further demonstrated that PtNP improved the ROS generation ability due to the oxygen supply from the decomposition of H_2_O_2_. Therefore, Pt NP could enhance the ROS production of CPT‐TK‐HPPH.

### 2.5 Light‐Activated Drug Release Behavior

The thioether bond in the prodrug has outstanding oxidation responsiveness, thus, we investigated the drug release behavior of the nanoparticles with 660 nm laser irradiation with and without H_2_O_2_. As presented in Figure 2F, the CPT‐TK‐HPPH/Pt NP and CPT‐CC‐HPPH/Pt NP without stimulation showed slow drug release behavior, and only 10% and 16% of the CPT‐CC‐HPPH/Pt NP and CPT‐TK‐HPPH/Pt NP released after 168 h, proving that the prodrug in the nanoparticle was barely released without stimulation. In contrast, when the nanoparticle was irradiated by a 660 nm laser, the CPT‐TK‐HPPH/Pt NP, which had a thioether bond released fast, revealed light‐activated drug release behavior. However, the release behavior of CPT‐CC‐HPPH/Pt NP without a thioether bond could not be accelerated even under 660 nm laser irradiation. Additionally, to verify whether H_2_O_2_ could accelerate the drug release, we estimated the drug release behavior of CPT‐TK‐HPPH NP, CPT‐CC‐HPPH/Pt NP, and CPT‐TK‐HPPH/Pt NP in the presence of 5 × 10^−3^ m H_2_O_2_, and the results revealed that CPT‐CC‐HPPH/Pt NP still showed slow drug release behavior and that the CPT in CPT‐TK‐HPPH/Pt NP exhibited a much faster release rate than that of CPT‐TK‐HPPH NP, demonstrating that the adding PtNP could accelerate the release behavior of the ROS‐responsive prodrug. We further studied the drug release behavior of CPT‐TK‐HPPH NP, CPT‐CC‐HPPH/Pt NP, and CPT‐TK‐HPPH/Pt NP in the presence of 5 × 10^−3^ m H_2_O_2_ and 660 nm laser irradiation. The release rate of the CPT‐TK‐HPPH NP and CPT‐CC‐HPPH/Pt NP was 86% and 32% within 168 h. As expected, the CPT‐TK‐HPPH/Pt NP demonstrated rapid CPT release, and ≈100% of the CPT was released after 168 h, which confirmed that CPT‐TK‐HPPH/Pt NP could realize light‐activated drug release, thus showing good behavior of on‐demand CPT release under 660 nm laser irradiation.

### 2.6 Cellular Uptake Efficiency

**Figure 3:**
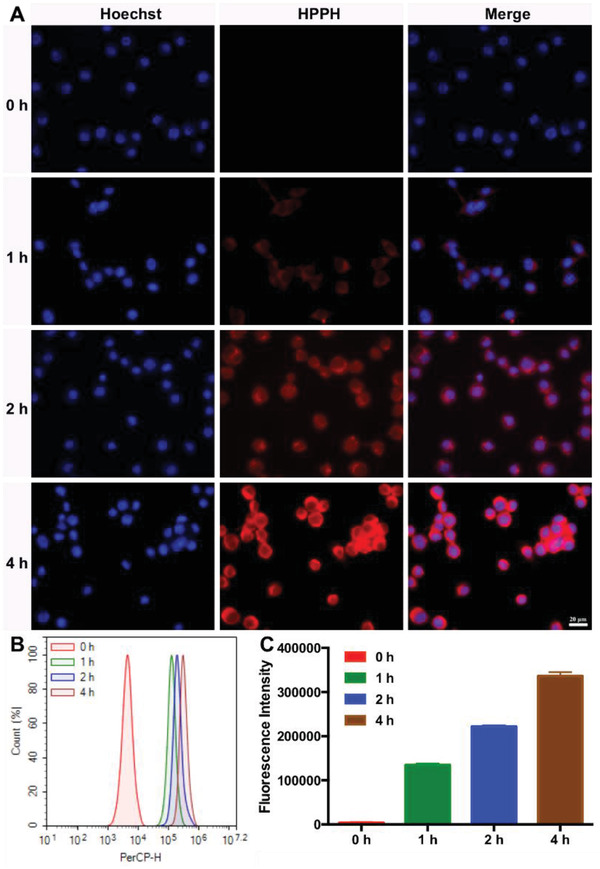
Cellular uptake ability of CT26 cells. A) Fluorescence images incubated with CPT‐TK‐HPPH/Pt NP from 0 to 4 h (blue channel: Hoechst, red channel: HPPH, scale bar: 20 µm). B) Flow cytometry analysis of cellular uptake ability. C) Quantitative analysis of fluorescence intensity. All quantitative data are presented as mean ± SD (*n* = 3).

Next, we evaluated the cellular uptake efficiency of CPT‐TK‐HPPH/Pt NP on mouse colon carcinoma cell line CT26 cells. The time‐dependent cellular uptake behavior of CPT‐TK‐HPPH/Pt NP was evident from 0 to 4 h, as shown in **Figure** **3**. After incubation for 1 h, we observed a dim red fluorescence signal in the HPPH column. Furthermore, a strong red fluorescence signal that circled around the blue cell nuclei, which were stained with Hoechst 33 342, was detected after 4 h incubation. This demonstrated that the CPT‐TK‐HPPH/Pt NP were internalized into the cells with a time‐dependent manner. Further, we determined the intracellular fluorescence signal of the CPT‐TK‐HPPH/Pt NP using flow cytometry. The results were consistent with the fluorescence images.

### 2.7 Intracellular ROS Level

**Figure 4:**
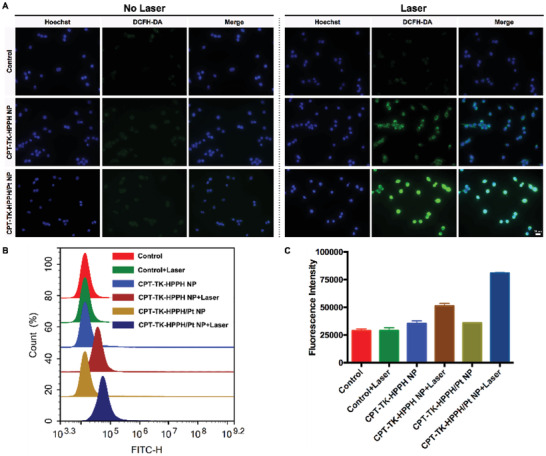
A) The fluorescence images of intracellular ROS generation using DCFH‐DA as the sensor (Blue channel: Hoechst, Green channel: DCFH‐DA, scale bar: 20 µm). B) The flow cytometry analysis and C) the quantitative analysis of intracellular ROS generation. All quantitative data are presented as mean ± SD (*n* = 3).

Encouraged by the in vitro ROS generation study, we used a fluorescence probe, 2′,7′‐dichlorofluorescin diacetate (DCFH‐DA), to detect the intracellular ROS level. DCFH‐DA did not have fluorescence itself, but the ROS in the cells could oxidize the non‐fluorescent DCFH into fluorescent DCF. As shown in **Figure** **4**A, the control group without treatment had little fluorescence and revealed no significant change under 660 nm laser irradiation. Moreover, a negligible fluorescence signal was observed in the groups of CPT‐TK‐HPPH NP and CPT‐TK‐HPPH/Pt NP without 660 nm laser irradiation. When the CPT‐TK‐HPPH NP and CPT‐TK‐HPPH/Pt NP were treated with 660 nm laser irradiation at a density of 200 mW cm^−2^ for 5 min, the DCFH‐DA channel, which showed strong intracellular fluorescence, demonstrated that CPT‐TK‐HPPH NP and CPT‐TK‐HPPH/Pt NP produced intracellular ROS for photodynamic therapy. Notably, the intracellular ROS level of CPT‐TK‐HPPH/Pt NP was higher than that of CPT‐TK‐HPPH NP because of the addition of PtNP, which supplied more oxygen for ROS generation. Additionally, we analyzed the quantitative data for the intracellular ROS level through flow cytometry, as shown in Figure 4B,C. The results proved that the ROS levels of CPT‐TK‐HPPH NP and CPT‐TK‐HPPH/Pt NP under 660 nm laser irradiation were 1.5‐ and 2.3‐fold higher than those of the no‐laser groups, which is consistent with the image data.

### 2.8 In Vitro Cytotoxicity Assay

**Figure 5:**
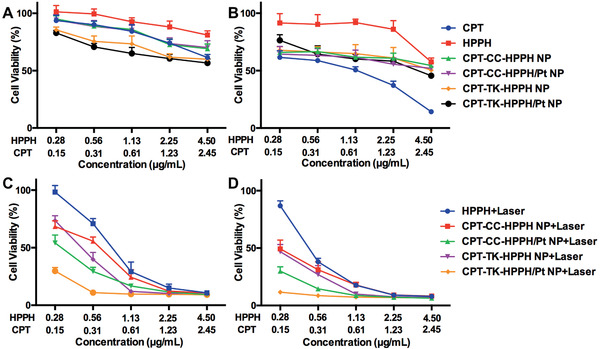
Cell viability of CT26 cells after treatment with CPT, HPPH, CPT‐CC‐HPPH NP, CPT‐CC‐HPPH/Pt NP, CPT‐TK‐HPPH NP, and CPT‐TK‐HPPH/Pt NP for A) 24 and B) 48 h. Cell viability of HPPH, CPT‐CC‐HPPH NP, CPT‐CC‐HPPH/Pt NP, CPT‐TK‐HPPH NP, and CPT‐TK‐HPPH/Pt NP with 660 nm laser irradiation for incubation for C) 24 and D) 48 h. All quantitative data are presented as mean ± SD (*n* = 6).

The cytotoxicity assays of the various nanoparticles with or without 660 nm laser irradiation were evaluated on CT26 cells using the 3‐(4,5‐dimethyl‐2‐thiazolyl)‐2,5‐diphenyl‐2‐H‐tetrazolium bromide (MTT) method. As presented in **Figure** **5**A,B, we first investigated the cytotoxicity assays of CPT, HPPH, CPT‐CC‐HPPH NP, CPT‐CC‐HPPH/Pt NP, CPT‐TK‐HPPH NP, and CPT‐TK‐HPPH/Pt NP cultured with CT26 cells for 24 and 48 h. These groups exhibited negligible cytotoxicity on CT26 cells after 24 h incubation, but CPT showed decreased cell viability in a dose‐dependent manner, which demonstrated that CPT could inhibit the proliferation of CT26 cells. However, other nanoparticle groups exhibited a limited cell inhibition after incubation for 48 h, which suggested that the intracellular release of CPT in CPT‐CC‐HPPH NP, CPT‐CC‐HPPH/Pt NP, CPT‐TK‐HPPH NP, and CPT‐TK‐HPPH/Pt NP was delayed without the 660 nm laser irradiation. What's more, the half‐maximal inhibitory concentration (IC50) is summarized in Figure S7 (Supporting Information). Further, we studied the cytotoxicity assay under 660 nm laser irradiation. The results are presented in Figure 5C,D, the HPPH, CPT‐CC‐HPPH NP, CPT‐CC‐HPPH/Pt NP, CPT‐TK‐HPPH NP, and CPT‐TK‐HPPH/Pt NP displayed apparent phototoxicity under 660 nm laser irradiation at a density of 200 mW cm^−2^ for 5 min. The cell viabilities of the aforementioned groups incubated for 48 h were 86.81%, 49.33%, 29.93%, 46.91%, and 11.64% with equivalent doses of CPT and HPPH of 0.28 and 0.15 µg mL^−1^, respectively, which demonstrated that CPT‐CC‐HPPH NP and CPT‐CC‐HPPH/Pt NP used with non‐responsive prodrug produced enough ROS to kill cells under 660 nm laser irradiation, but the cytotoxicity was not as effective as that of CPT‐TK‐HPPH NP and CPT‐TK‐HPPH/Pt NP, which further proved that CPT‐TK‐HPPH NP and CPT‐TK‐HPPH/Pt NP produced enough ROS to kill the cells and accelerated the release of CPT to attain effective chemophotodynamic therapy for colon cancer. Notably, for both the CPT‐CC‐HPPH/Pt NP group and CPT‐TK‐HPPH/Pt NP groups, when PtNP was added, the cytotoxic effects were higher than those for the CPT‐TK‐HPPH NP and CPT‐CC‐HPPH NP groups under 660 nm laser irradiation. This further demonstrated that the PtNP could produce oxygen and significantly enhance the photodynamic efficiency of the nanoparticles.

### 2.9 Cell Apoptosis Analysis

**Figure 6:**
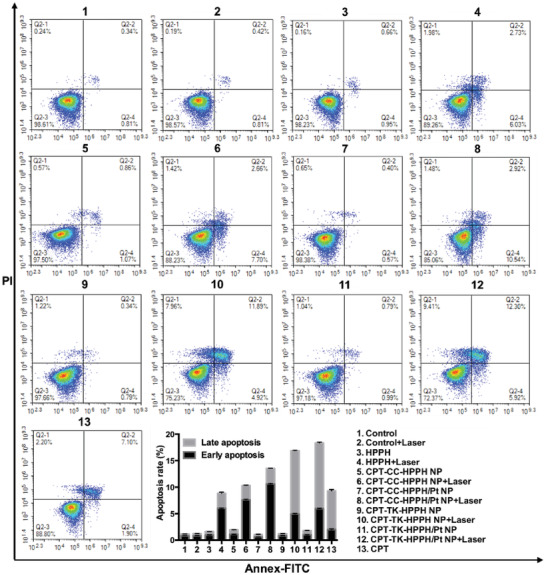
Flow cytometric analysis of CT26 cell apoptosis induced by different treatments. All quantitative data are presented as mean ± SD (*n* = 3).

We evaluated the cell apoptosis of CPT, HPPH, CPT‐CC‐HPPH NP, CPT‐CC‐HPPH/Pt NP, CPT‐TK‐HPPH NP, and CPT‐TK‐HPPH/Pt NP with and without 660 nm laser irradiation using an Annexin V‐FITC apoptosis detection kit. As shown in **Figure** **6**, the apoptotic ratios of CPT‐CC‐HPPH NP, CPT‐CC‐HPPH/Pt NP, CPT‐TK‐HPPH NP, and CPT‐TK‐HPPH/Pt NP treated with 660 nm laser irradiation were higher than those of the group that was not irradiated, which suggested that the laser could remarkably enhance the antitumor effects of the nanoparticles. Notably, the CPT‐TK‐HPPH/Pt NP group treated with 660 nm laser irradiation exhibited the highest apoptotic ratio (18.12%) than that of other groups, which was consistent with the in vitro cytotoxicity assay, implying that the light‐triggered release behavior of CPT and the enhanced ROS production ability of PtNP in CPT‐TK‐HPPH/Pt NP improved chemophotodynamic therapy for colon cancer.

### 2.10 In Vivo Imaging Study

**Figure 7:**
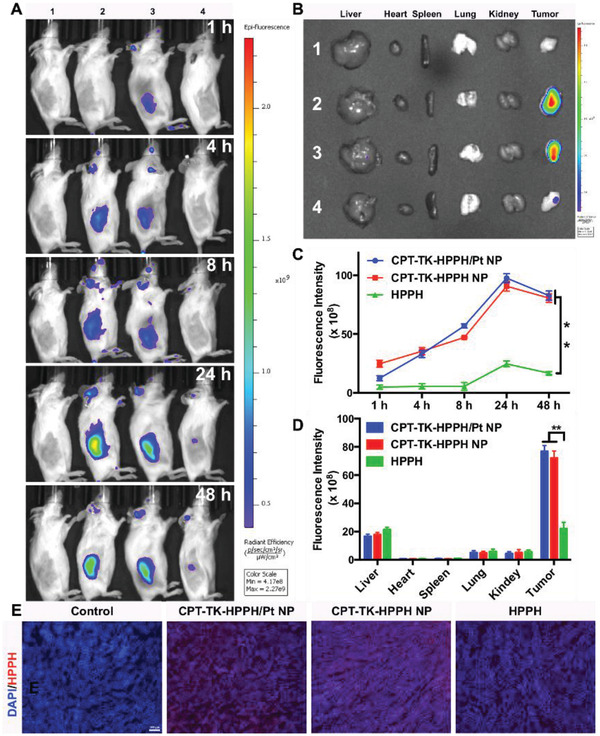
A) In vivo time‐dependent fluorescence image of tumors in CT26 tumor‐bearing mice. B) In vitro imaging of the livers, hearts, spleens, lungs, kidneys, and tumors excised from CT26 tumor‐bearing mice after 48 h (1. Control group, 2. CPT‐TK‐HPPH/Pt NP, 3. CPT‐TK‐HPPH NP, 4. HPPH). Quantitative fluorescence intensity of C) in vivo and ex vivo tumor tissues measured by RIO value. D) Fluorescence images of ex vivo tissues (blue channel: DAPI, red channel: HPPH, scale bar: 100 µm). All quantitative data are presented as mean ± SD (*n* = 3). “**” means the *P* < 0.01.

Whether a drug accumulates at the tumor sites directly affects the antitumor effect in vivo, so we evaluated the biodistribution and tumor targeting ability of CPT‐TK‐HPPH/Pt NP using near‐infrared (NIR) fluorescence imaging and photoacoustic (PA) imaging of CT26 tumor‐bearing BALB/C mice. In the NIR fluorescence imaging shown in **Figure** **7**A, the fluorescence of CPT‐TK‐HPPH NP and CPT‐TK‐HPPH/Pt NP at the tumor sites gradually increased from 1 to 24 h, and the highest fluorescence signal of CPT‐TK‐HPPH/Pt NP at tumor sites was observed at 24 h postinjection, demonstrating that the CPT‐TK‐HPPH/Pt NP had effective tumor accumulation ability and that the DSPE‐PEG enhanced the blood circulation time and EPR effect of the CPT‐TK‐HPPH/Pt NP, which may improve the in vivo antitumor efficiency. Besides, the fluorescence signal of HPPH at the tumor sites was lower than those of CPT‐TK‐HPPH NP and CPT‐TK‐HPPH/Pt NP because of the poor tumor target ability of HPPH. Additionally, we explored the fluorescence intensity of the major organs at 48 h. The results (Figure 7B) showed that, in addition to the tumor sites, the nanoparticles were mainly distributed in the liver tissues, suggesting that the nanoparticles were mainly metabolized by the liver. The quantitative fluorescence intensity of the tumors in in vivo and ex vivo tissues measured by their RIO value were demonstrated in Figure 7C,D. Moreover, we investigated the distribution of the nanoparticles in tumor tissue. As seen in Figure 7E, the CPT‐TK‐HPPH/Pt NP exhibited strong red fluorescence, which was consistent with the above results, further indicating that the CPT‐TK‐HPPH/Pt NP was effectively accumulated at the tumor sites.

**Figure 8:**
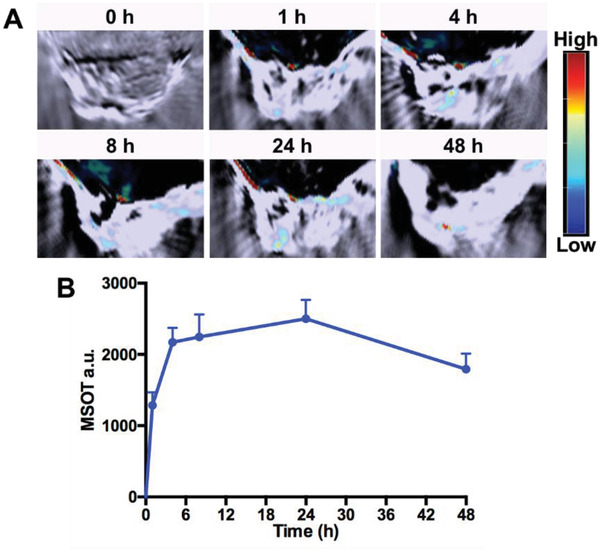
A) In vivo PA imaging of CT26 tumor‐bearing mice before and after injection with CPT‐TK‐HPPH/Pt NP. B) MSOT intensity variation of tumor tissues at 680 nm. All quantitative data are presented as mean ± SD (*n* = 3).

For the PA imaging study, we investigated the PA signal of the CPT‐TK‐HPPH/Pt NP with different concentrations in vitro using a multispectral optoacoustic tomography (MSOT) small animal scanner. The results shown in Figure S8 (Supporting Information) demonstrated that the CPT‐TK‐HPPH/Pt NP had good linear correlation with the concentration in the range of 1.4–45 µg mL^−1^, which further indicated that CPT‐TK‐HPPH/Pt NP has great potential in PA imaging. Thereafter, we evaluated the PA signals of CPT‐TK‐HPPH/Pt NP at tumor sites in CT26 tumor‐bearing mice, as shown in **Figure** **8**, there was a pronounced PA signal for CPT‐TK‐HPPH/Pt NP at the tumor sites postinjection. The PA signal also increased from 1 to 24 h, suggesting that the CPT‐TK‐HPPH/Pt NP could accumulate at the tumor sites because the DSPE‐PEG improved the blood circulation capacity and enhanced the internalization efficiency.

### 2.11 Pharmacokinetic Study

Besides, we investigated the blood circulation time of CPT and CPT‐TK‐HPPH/Pt NP by pharmacokinetic study. As shown in Figure S9 (Supporting Information), the CPT in plasma removed quickly, and only 5.58% of CPT was left in plasma at 8 h. While the CPT‐TK‐HPPH/Pt NP exhibited the prolonged circulation times, and were 17‐fold higher than those of the CPT at 24 h, showing that the CPT‐TK‐HPPH/Pt NP has longer half‐life time and better long‐circulating time than CPT.

### 2.12 In Vivo Antitumor Study

**Figure 9:**
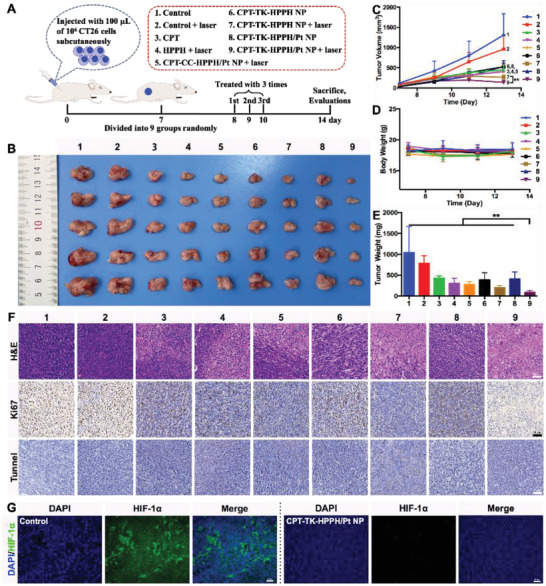
A) Schematic illustration of in vivo antitumor efficacy of CPT‐TK‐HPPH/Pt NP, B) photograph, C) growth curves, D) body weight, and E) tumor weight of CT26 tumor‐bearing mice in each group. F) Representative H&E stained images, K_i_‐67 immune histochemical images, and tunnel histochemical images of CT26 tumors (scale bar: 50 µm). G) Representative immune‐fluorescence imaging of situation of hypoxia in tumors (scale bar: 50 µm). All quantitative data are presented as mean ± SD (*n* = 5). “**” means the *P* <0.01. (1. Control, 2. Control+Laser, 3. CPT, 4. HPPH+Laser, 5. CPT‐CC‐HPPH/Pt NP+Laser, 6. CPT‐TK‐HPPH NP, 7. CPT‐TK‐HPPH NP+Laser, 8. CPT‐TK‐HPPH/Pt NP, 9. CPT‐TK‐HPPH/Pt NP+Laser.).

Inspired by the in vitro cytotoxicity assay and the in vivo imaging study, the CT26 tumor‐bearing BALB/C mice were selected to investigate the antitumor ability of the polymeric nanoparticles. As shown in **Figure** **9**C, the tumor volume of the mice treated with 1) the control group and 2) the control + laser group grew quickly, demonstrating that the 660 nm irradiation at the tumor sites could not inhibit the tumors. Furthermore, the tumor treated with the 3) CPT and 4) HPPH+laser groups could be smaller, but the antitumor effect was unsatisfactory, implying that chemotherapy or photodynamic therapy alone were insufficient to inhibit tumor growth. The tumor inhibitory effect of the (5) CPT‐CC‐HPPH/Pt NP+laser group was similar to that of the 3) CPT and 4) HPPH+laser groups because the CPT in CPT‐CC‐HPPH/Pt NP could not be released under 660 nm laser irradiation, thus only showing the effect of photodynamic therapy. In addition, the tumor inhibition ability of the 6) CPT‐TK‐HPPH NP and 8) CPT‐TK‐HPPH/Pt NP groups was not better than that of the 7) CPT‐TK‐HPPH NP+laser and 9) CPT‐TK‐HPPH/Pt NP+laser groups, which further exhibited that 660 nm laser irradiation accelerated the release of CPT in the ROS‐responsive prodrug to attain synergistic chemophotodynamic therapy. Notably, the growth inhibition ability of the 9) CPT‐TK‐HPPH/Pt NP+laser group was significantly higher than that of other groups owing to the PtNP in the CPT‐TK‐HPPH/Pt NP catalyzing the decomposition of H_2_O_2_ in tumor tissue to produce oxygen and ameliorate tumor hypoxia. This enhances the photodynamic therapy effect and is synergistic with chemotherapy for colon cancer treatment. The photographs of the tumors (Figure 9B) and tumor weight (Figure 9E) were consistent with the growth curves. The lack of noticeable body weight changes is shown in Figure 9D, demonstrating that the aforementioned group induced no serious side effects on the health of the mice. Furthermore, we investigated the hematoxylin and eosin (H&E) images of major organ tissues harvested from all groups at the end of the in vivo antitumor study. As shown in Figure S10 (Supporting Information), we observed the cytoplasmic loosening in the liver tissues of the 3) CPT group, which may be due to the toxicity of the CPT. No pronounced histopathological damage was exhibited in other organ tissues, demonstrating that the CPT‐TK‐HPPH/Pt NP had good biocompatibility in colon cancer therapy.

Next, we evaluated the proliferation and apoptosis of tumor cells via H&E staining, K_i_‐67 staining, and tunnel staining. As shown in Figure 9F, necrotic tumor cells were evident in the 9) CPT‐TK‐HPPH/Pt NP+laser group, and the significant proliferation and apoptosis of tumor cells in the 9) CPT‐TK‐HPPH/Pt NP+laser group were also revealed by K_i_‐67 staining and tunnel staining. The quantitative data of K_i_‐67 LI and apoptotic index of tumors in each group demonstrated in Figures S11 and S12 (Supporting Information). Taken together, the CPT‐TK‐HPPH/Pt NP has great potential for inhibiting tumor cell proliferation via enhanced chemophotodynamic therapy, which concurs with the results of the in vivo antitumor study.

Furthermore, we evaluated the situation of hypoxia in the tumors using immune‐fluorescence staining methods. As shown in Figure 9G, the 4',6‐diamidino‐2‐phenylindole (DAPI) channel, with blue fluorescence, represented the cell nuclei, and the HIF‐1*α* channel, with green fluorescence, represented tumor hypoxia. Pronounced green fluorescence was observed in the 1) control group, demonstrating that the tumor was hypoxic without the treatment. In contrast, the CPT‐TK‐HPPH/Pt NP significantly alleviated tumor hypoxia because the PtNP in CPT‐TK‐HPPH/Pt NP decomposed the H_2_O_2_ in tumor tissue and produce oxygen. The results proved that the CPT‐TK‐HPPH/Pt NP is a promising candidate for relieving hypoxia in photodynamic therapy.

## 3 Conclusion

In summary, we developed a small platinum nanozyme (PtNP) loaded ROS‐responsive prodrug nanoparticle (CPT‐TK‐HPPH/Pt NP) to attain enhanced chemophotodynamic synergistic therapy for colon cancer. In this nanoparticle, the PtNP could decompose H_2_O_2_ into oxygen, leading to improvement of ROS generation ability of HPPH. The release of CPT could be controlled by 660 nm laser irradiation owing to the thioketal cleavage in the presence of ROS. Under the exposure of 660 nm laser irradiation, CPT‐TK‐HPPH/Pt NP displayed enhanced cell cytotoxicity and inhibition of cell proliferation. The fluorogenicity nature of HPPH enabled us to use the CPT‐TK‐HPPH/Pt NP as an outstanding probe to visualize cellular uptake in vitro, and tissue distribution in vivo via fluorescence imaging and photoacoustic imaging, which demonstrated the promoting accumulation of CPT‐TK‐HPPH/Pt NP in tumor sites. Notably, the CPT‐TK‐HPPH/Pt NP could effectively inhibit tumor growth in vivo through the combined chemotherapy and photodynamic therapy. The CPT‐TK‐HPPH/Pt NP with excellent “smart” drug release characteristic and enhanced PDT efficiency would provide a new opportunity for clinical treatment of colon cancer.

## 4 Experimental Section

##### Materials

2‐(1‐hexyloxyethyl)‐2‐devinyl pyropheophorbide‐a (HPPH) was purchased from Shanghai AZBIOCHEM Co., Ltd. Chloroplatinic acid hexahydrate (H_2_PtCl_6_·6H_2_O), polyvinyl alcohol (PVA), sodium borohydride (NaBH_4_), and 1,6‐hexanediol were obtained from Sigma‐Aldrich Company. DSPE‐PEG_2000_ were purchased from Shanghai Aladdin Biochemical Technology Co., Ltd. 3,3′‐[(1‐methylethylidene)bis(thio)]bis‐Propanoic acid was obtain from Beijing HWRK CHEM Co., Ltd. CPT was purchased from Meilun Biology Technology Co., Ltd. TMB and H_2_O_2_ were obtained from Shanghai Titanchem Co., Ltd. Reactive Oxygen Species Assay Kit and Hoechst 33 342 were purchased from Beyotime Biotechnology Co., Ltd. Annexin V‐FITC Apoptosis Detection Kit (BD Pharmingen) was obtained from Shanghai Univ Biotechnology Co., Ltd. All the materials used in this study were analytic grade and used as received.

##### Synthesis and Characterization of the Prodrug

In this part, the ROS‐responsive prodrug, CPT‐TK‐HPPH, was synthesized using CPT, HPPH, and an ROS‐responsive linker. The prodrug, CPT‐CC‐HPPH, without an ROS‐responsive linker, was used as the control group. As shown in Figure S1 (Supporting Information), compound **2** which contained two hydroxyl groups, was obtained from the reduction of the corresponding carboxyl residues in 3,3′‐[(1‐methylethylidene)bis(thio)]bis‐propanoic acid (compound **1**) by NaBH_4_. The CPT drug was conjugated into one hydroxyl group in compound **2** or compound **4** (1,6‐hexanediol) by reaction with stoichiometric phosgene in the presence of the catalyst DMAP. The esterification of the hydroxyl group in compound **3** or compound **5** with HPPH to obtain CPT‐TK‐HPPH or CPT‐CC‐HPPH. Thereafter, the prodrugs and the concentrations of CPT and HPPH were characterized using ^1^H NMR spectra (Varian 400 spectrometer, Varian, USA), high performance liquid chromatography (HPLC), and UV–vis spectroscopy (CPT‐TK‐HPPH: ^1^H NMR (400 MHz, Chloroform‐d) *δ* 9.80 (d, J = 13.5 Hz, 1H), 9.51 (s, 1H), 8.44 (d, J = 10.1 Hz, 1H), 8.01 (dd, J = 12.5, 8.5 Hz, 1H), 7.63 (d, J = 5.2 Hz, 2H), 7.51–7.13 (m, 5H), 5.91 (dd, J = 10.7, 6.7 Hz, 1H), 5.68 (d, J = 17.2 Hz, 1H), 5.41–5.28 (m, 2H), 5.21–5.09 (m, 1H), 5.07–4.95 (m, 1H), 4.80 (dd, J = 38.6, 18.8 Hz, 1H), 4.47–4.39 (m, 1H), 4.28–4.15 (m, 3H), 4.15–4.04 (m, 2H), 3.72 (dt, J = 14.0, 6.9 Hz, 3H), 3.63 (d, J = 2.3 Hz, 1H), 3.35 (d, J = 5.7 Hz, 3H), 3.28 (s, 3H), 2.64 (tq, J = 7.0, 2.3 Hz, 3H), 2.56 (tdd, J = 7.3, 5.2, 2.5 Hz, 3H), 2.37 (d, J = 12.0 Hz, 2H), 2.25–2.03 (m, 6H), 1.92 (dd, J = 8.2, 4.8 Hz, 2H), 1.87–1.60 (m, 15H), 1.49 (dd, J = 3.9, 2.2 Hz, 8H), 0.96 (td, J = 7.5, 2.5 Hz, 4H), 0.77 (td, J = 6.9, 3.8 Hz, 3H). CPT‐CC‐HPPH: ^1^H NMR (400 MHz, Chloroform‐d) *δ* 9.78 (d, J = 5.3 Hz, 1H), 9.52 (s, 1H), 8.50 (s, 1H), 8.14–7.97 (m, 2H), 7.76–7.59 (m, 2H), 7.51 (d, J = 7.3 Hz, 1H), 7.27 (d, J = 5.3 Hz, 3H), 5.91 (dd, J = 6.7, 3.9 Hz, 1H), 5.68 (d, J = 17.2 Hz, 1H), 5.37 (d, J = 17.3 Hz, 1H), 5.23 (d, J = 19.8 Hz, 1H), 5.14–5.03 (m, 3H), 4.46 (d, J = 7.5 Hz, 1H), 4.28 (d, J = 8.6 Hz, 1H), 4.12–3.99 (m, 2H), 3.98–3.82 (m, 2H), 3.78–3.56 (m, 8H), 3.37 (s, 3H), 3.27 (s, 3H), 2.65 (dtd, J = 13.3, 7.0, 3.5 Hz, 1H), 2.49 (m, 1H), 2.25 (m, 4H), 2.12 (dd, J = 6.7, 1.8 Hz, 4H), 1.80 (dd, J = 7.3, 1.6 Hz, 4H), 1.72 (q, J = 7.7, 6.1 Hz, 6H), 1.59 (d, J = 10.7 Hz, 9H), 1.44 (h, J = 6.8 Hz, 5H), 1.37–1.24 (m, 13H), 1.22 (d, J = 6.8 Hz, 8H), 0.98 (t, J = 7.5 Hz, 3H), 0.77 (dt, J = 6.9, 3.4 Hz, 3H)).

##### Preparation and Characterization of Platinum Nanozyme

PtNP was synthesized using a reduction method with sodium borohydride as reported.^[^ ^14c^ ^]^ In detail, 1 mL of H_2_PtCl_6_·6H_2_O (10 × 10^−3^ m) and PVP (16 mg) were dissolved in 9 mL water, and then an ice‐cold NaBH_4_ solution (4.4 mg) was added under stirring for 4 h at room temperature. At last, the PtNP was obtained via a 100 kDa MWCO electrocatalyst. The particle size, morphology, and concentration of the PtNP were characterized using DLS, TEM, and inductively coupled plasma‐mass spectrometry (ICP‐MS). Meanwhile, the cell viability of the PtNP on mouse fibroblast cell line 3T3 cells using the MTT method was studied. What's more, the oxygen production ability of PtNP through the typical TMB colorimetric reaction was further investigated.^[^ ^14b^ ^]^ Briefly, different concentrations of PtNP were added into a mixture of H_2_O_2_ (10 × 10^−3^ m) and TMB (1 × 10^−3^ m), and UV–vis spectroscopy was used to measure the absorbance of the mixture at 652 nm over a determined time.

##### Preparation and Characterization of Polymeric Nanoparticle

The polymeric nanoparticle was prepared through a double emulsion method.^[^ ^16^ ^]^ First, 200 µg of the prodrug CPT‐TK‐HPPH was dissolved in 2 mL of dichloromethane, and 15 µg of PtNP was sonicated via a probe‐type ultrasonic processor for 6 min to obtain the primary emulsion. Additionally, the as‐prepared primary emulsion was added into 10 mL of DSPE‐PEG solution (0.2 mg mL^−1^) and sonicated for another 6 min. The prodrug CPT‐TK‐HPPH and PtNP co‐loaded nanoparticle (CPT‐TK‐HPPH/Pt NP) was obtained after removing the organic solvent and the unloaded agents using rotary evaporation under vacuum and then filtering. The CPT‐TK‐HPPH loaded nanoparticle (CPT‐TK‐HPPH NP), the CPT‐CC‐HPPH loaded nanoparticle (CPT‐CC‐HPPH NP), and the CPT‐CC‐HPPH and PtNP co‐loaded nanoparticle (CPT‐CC‐HPPH/Pt NP) were prepared using the same method. The concentrations of CPT and HPPH loaded in the nanoparticles were calculated by its standard curve (Figure S2, Supporting Information) by HPLC and UV, respectively. And PtNP in the nanoparticles were investigated using ICP‐MS. The particle size, zeta potential, and morphology of the polymeric nanoparticles were determined by DLS and TEM.

##### ROS Generation In Vitro

The ROS generation of the polymeric nanoparticle exposed to 660 nm laser irradiation at 200 mW cm^−2^ was evaluated by measuring the absorbance changes of ABDA at 378 nm.^[^ ^15^ ^]^ In details, 100 µL of a 1 mg mL^−1^ ABDA solution was added to HPPH, CPT‐CC‐HPPH NP, CPT‐CC‐HPPH/Pt NP, CPT‐TK‐HPPH NP, and CPT‐TK‐HPPH/Pt NP solutions (equivalent to 10 µg mL^−1^ of HPPH), which were deoxygenated and contained 100 × 10^−6^ m H_2_O_2_. Thereafter, the aforementioned solutions were exposed to 660 nm laser irradiation at 200 mW cm^−2^. The ROS generation of the polymeric nanoparticle was evaluated by UV–vis spectroscopy at a preset time point.

##### Light‐Activated Drug Release Behavior

The light‐activated drug release behavior of the polymeric nanoparticle was investigated using a modified dialysis method in the presence of 660 nm laser irradiation and/or H_2_O_2_.^[^ ^17^ ^]^ Briefly, 1 mL of CPT‐CC‐HPPH/Pt NP, CPT‐TK‐HPPH NP, and CPT‐TK‐HPPH/Pt NP were sealed in dialysis bags (molecular mass cut off of 3500), and then incubated in 15 mL of phosphate buffer saline (PBS) containing 0 × 10^−3^ m, 5 × 10^−3^ m H_2_O_2_ with gentle shaking (100 rpm) at 37 °C. At predetermined time points, the laser group was placed under 660 nm laser irradiation at 200 mW cm^−2^ and then the medium for all groups was collected and replaced with prewarmed fresh PBS. The CPT was quantified by HPLC.

##### Cellular Uptake Efficiency

The cellular uptake ability of the CPT‐TK‐HPPH/Pt NP was investigated using mouse colon carcinoma cell line CT26 cells.^[^ ^18^ ^]^ CT26 cells were seeded into a 6‐well plate with a density of 2 × 10^5^. After incubation for 24 h, 2 mL of CPT‐TK‐HPPH/Pt NP (equivalent to 1 µg mL^−1^ of HPPH) was added, and the plates were incubated for another 1, 2, and 4 h. The cells were then washed with PBS three times, and the nuclei of the cells were stained using Hoechst 33 342 (10 µg mL^−1^) prior to fluorescence microscope observation. The quantitative data of the cellular uptake efficiency were also investigated using flow cytometry.

##### Intracellular ROS Level

DCFH‐DA^[^ ^19^ ^]^ was utilized as a probe to detect the intracellular ROS level of CPT‐TK‐HPPH NP and CPT‐TK‐HPPH/Pt NP. First, CT26 cells with a density of 1 × 10^5^ were seeded into a 12‐well plate and incubated for 24 h. Thereafter, 1 mL of CPT‐TK‐HPPH NP and CPT‐TK‐HPPH/Pt NP (equivalent to 1 µg mL^−1^of HPPH) were added, and the free medium was used as the control group. After incubation for 4 h, the DCFH‐DA (10 × 10^−6^ m) was added into each well and incubated for another 20 min. For the laser group, the medium was replaced by a fresh medium and placed under 660 nm laser irradiation at 200 mW cm^−2^ for 5 min. The cells were fixed with 70% EtOH and stained with Hoechst 33 342 (10 µg mL^−1^) before fluorescence microscope observation. Moreover, flow cytometry was used to collect the quantitative data about the intracellular ROS level.

##### In Vitro Cytotoxicity Assay

The cell cytotoxicity assay was investigated using the MTT method.^[^ ^20^ ^]^ In short, a density of 4 × 10^3^ CT26 cells was seeded into a 96‐well plate for 24 h. CPT, HPPH, CPT‐CC‐HPPH NP, CPT‐CC‐HPPH/Pt NP, CPT‐TK‐HPPH NP, and CPT‐TK‐HPPH/Pt NP with various concentrations were added and cultured for 12 h. The cells were treated with or without 660 nm laser irradiation at 200 mW cm^−2^ for 5 min as designed. The cells were then incubated for 12 and 36 h following the standard MTT method.

##### Cell Apoptosis Analysis

CT26 cells were seeded into a 6‐well plate with a density of 2 × 10^5^ and cultured for 24 h, then the CPT, HPPH, CPT‐CC‐HPPH NP, CPT‐CC‐HPPH/Pt NP, CPT‐TK‐HPPH NP, and CPT‐TK‐HPPH/Pt NP were added. After 12 h incubation, the cells were treated with or without 660 nm laser irradiation at 200 mW cm^−2^ for 5 min as designed and were incubated for another 12 h. Thereafter, cells in each group were collected and stained using the Annexin V‐FITC apoptosis detection kit^[^ ^21^ ^]^ according to the procedure given by the manufacture.

##### In Vivo Imaging Study

Animal experiments were approved by the Animal Ethics Committee of Sichuan University (SKLB2019). CT26 tumor‐bearing BALB/c mice were used to estimate the biodistribution and tumor targeting ability of CPT‐TK‐HPPH/Pt NP using NIR fluorescence imaging and PA imaging.^[^ ^22^ ^]^ When the tumor size was parable, the CPT‐TK‐HPPH/Pt NP was injected intravenously at an identical HPPH dose of 25 µg per mice, and CPT‐TK‐HPPH NP and HPPH were evaluated as control groups. An IVIS Lumina III imaging system (*E*x = 640 nm, *E*m = 670 nm) was then used to acquire fluorescence imaging at 1, 4, 8, 24, and 48 h postinjection. Next, the mice were sacrificed, and major organs were collected for ex vivo fluorescence imaging. Finally, the distribution of nanoparticles in tumor tissue was evaluated for different sections.

An MSOT small animal scanner was used to investigate the PA signal of CPT‐TK‐HPPH/Pt NP with different concentrations in vitro at 680 nm. The tumor targeting ability of the CPT‐TK‐HPPH/Pt NP on CT26 tumor‐bearing BALB/c mice at 1, 4, 8, 24, and 48 h postinjection was then evaluated.

##### Pharmacokinetic Study

The pharmacokinetic of CPT and CPT‐TK‐HPPH/Pt NP were carried on the Sprague Dawley (SD) rats (200 g). Briefly, the CPT or CPT‐TK‐HPPH/Pt NP was injected intravenously at the dose of 3 mg kg^−1^ of CPT. Then collected the blood sample through eye puncture at 5, 15, 30 min, 1, 2, 4, 8, 24, and 48 h postinjection. All of the blood samples were centrifugated to obtain plasma. 10‐Hydroxycamptothecin and methanol was used as internal standard and extractant, respectively. Finally, the plasma samples were measured by HPLC Agilent 1260 Infinity system. CPT and CPT‐TK‐HPPH/Pt NP with the equal dose in plasma was used as 100%.

##### In Vivo Antitumor Study

As shown in Figure 9A, CT26 cells with a density of 1 × 10^6^ were injected subcutaneously in the right leg of the BALB/c mice. When the tumor volume reached ≈100 mm^3^, the mice were divided randomly into nine groups (five mice per group): 1) control group (without treatment); 2) control+laser group, in which the mice were irradiated by a 660 nm laser at 200 mW cm^−2^ for 5 min at the tumor sites; 3) CPT group (3 mg kg^−1^); 4) HPPH+laser group (5.5 mg kg^−1^); 5) CPT‐CC‐HPPH/Pt NP+laser group; 6) CPT‐TK‐HPPH NP group; 7) CPT‐TK‐HPPH NP+laser group; 8) CPT‐TK‐HPPH/Pt NP group; 9) CPT‐TK‐HPPH/Pt NP+laser group (the doses received by groups (5–9) were equivalent to 3 mg kg^−1^ of CPT and 5.5 mg kg^−1^ HPPH). Group (3), (6), and (8) were treated with the aforementioned drug intravenously through the tail vein. Groups (4), (5), (7), and (9) were intravenously injected with the aforementioned drug and placed under 660 nm laser irradiation at 200 mW cm^2^ for 5 min. The respective treatment was provided to mice from all groups three times, and the tumor volume and weight of the mice were measured every other day. Finally, the mice were sacrificed and the tumor and major organ tissue of all groups for hematoxylin and eosin (H&E) staining were collected.^[^ ^23^ ^]^ Moreover, the proliferation and apoptosis of tumor cells via K_i_‐67 staining and tunnel staining were evaluated.^[^ ^24^ ^]^ The tumor hypoxia situation was investigated via immune‐fluorescence staining.^[^ ^25^ ^]^

##### Statistical Analysis

All data were presented as the mean value ± standard deviation (SD). The statistical analysis was performed using Prism 6.0 software. Student's *t*‐test or one‐way analysis of variance (ANOVA) was used for statistical analysis. *P* < 0.05 or *P* < 0.01 marked with “*” or “**” were considered to be statistically significant.

## Conflict of Interest

The authors declare no conflict of interest.

## Supporting information

Supporting Information
