# Supplementary material for: Polymeric Nanoparticles with ROS‐Responsive Prodrug and Platinum Nanozyme for Enhanced Chemophotodynamic Therapy of Colon Cancer
Source: Adv Sci (Weinh). 2020 Sep 6;7(20):2001853. doi: 10.1002/advs.202001853 (PMC7578901; doi:10.1002/advs.202001853)
Supplement: Supplementary file 1 — Supporting Information [file ADVS-7-2001853-s001.pdf]

© 2020 Wiley-VCH GmbH

ADVANCED SCIENCE
Open Access

Supporting Information

for *Adv. Sci.,* DOI: 10.1002/advs.202001853

Polymeric Nanoparticles with ROS-Responsive Prodrug and Platinum Nanozyme for Enhanced Chemophotodynamic Therapy of Colon Cancer

*Ying Hao, Yuwen Chen, Xinlong He, Yongyang Yu, Ruxia Han, Yang Li, Chengli Yang, Danrong Hu, and Zhiyong Qian**

Polymeric Nanoparticles with ROS-Responsive Prodrug and Platinum Nanozyme for Enhanced Chemo-Photodynamic Therapy of Colon Cancer

Ying Hao,[†] Yuwen Chen,[†] Xinlong He,[†] Yongyang Yu,[‡] Ruxia Han,[†] Yang Li,[†] Chengli Yang,[†] Danrong Hu,[†] Zhiyong Qian*,[†]

[†]State Key Laboratory of Biotherapy and Cancer Center, West China Hospital, Sichuan University, and Collaborative Innovation Center of Biotherapy, Chengdu, 610041, PR China

[‡]Department of Gastrointestinal Surgery, West China Hospital, Sichuan University, Chengdu, 610041, PR China

[*] To whom should be corresponded, Tel/Fax: +86-28-85557632, E-mail: anderson-qian@163.com (Qian ZY).

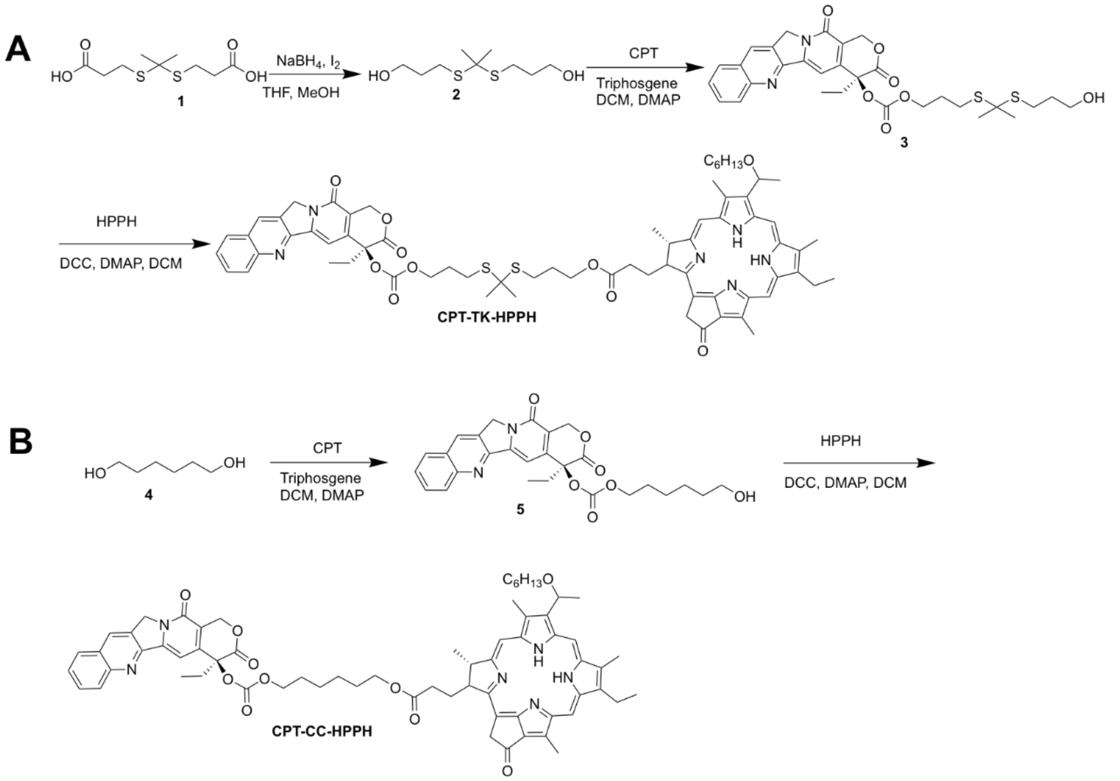

Figure S1. Synthesis method for (A) CPT-TK-HPPH and (B) CPT-CC-HPPH.

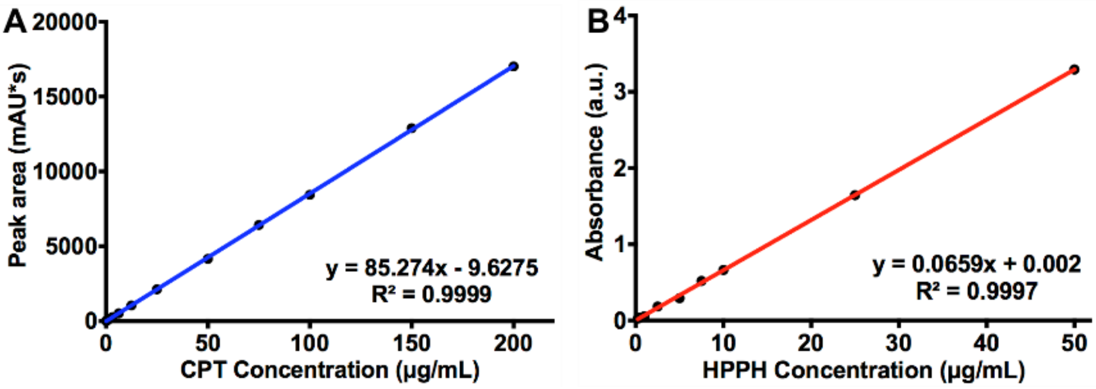

Figure S2. Linear correlation of (A) CPT and (B) HPPH against its corresponding concentrations (inset: the linear fitting equation).

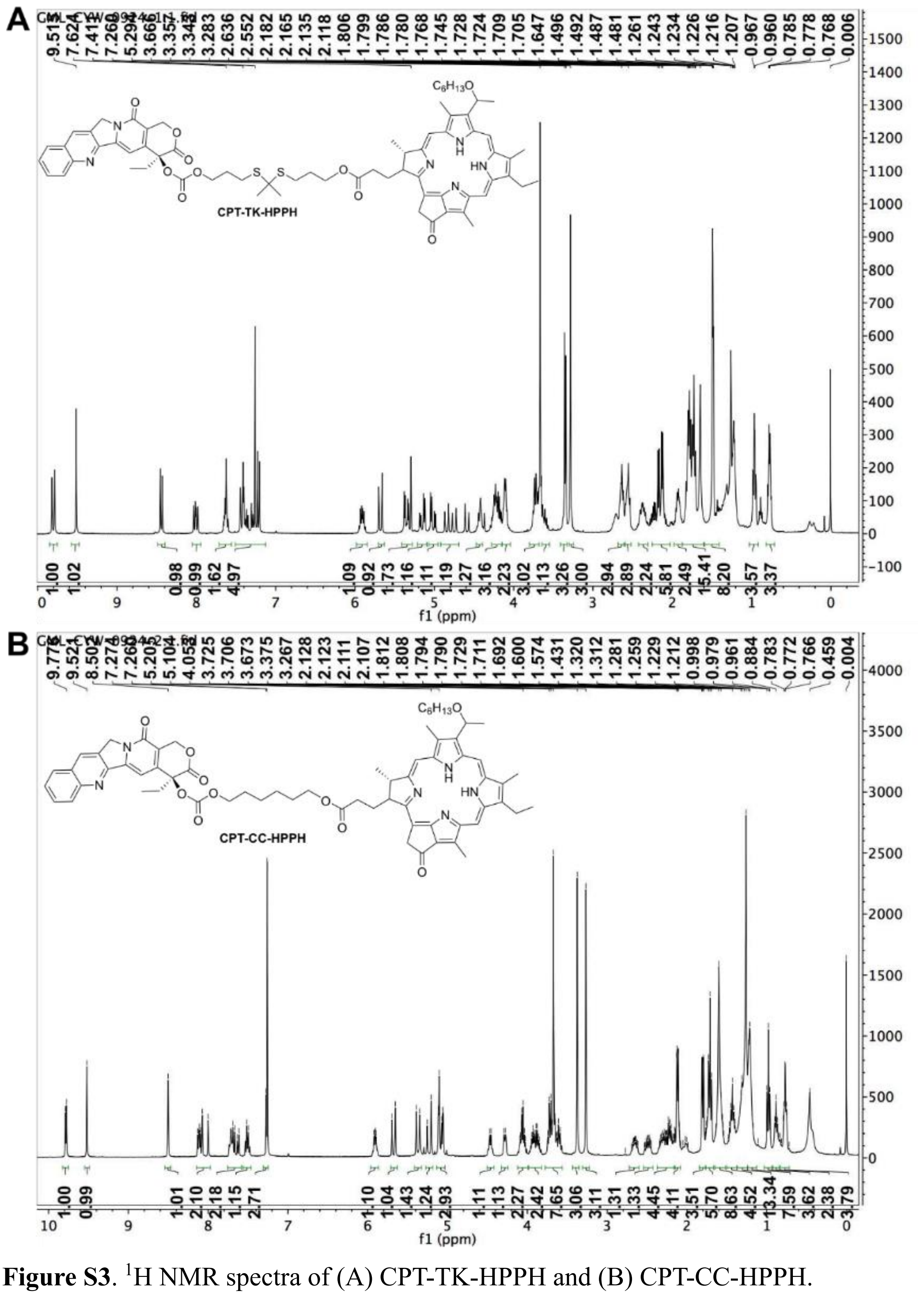

Figure S3. ^{1}H NMR spectra of (A) CPT-TK-HPPH and (B) CPT-CC-HPPH.

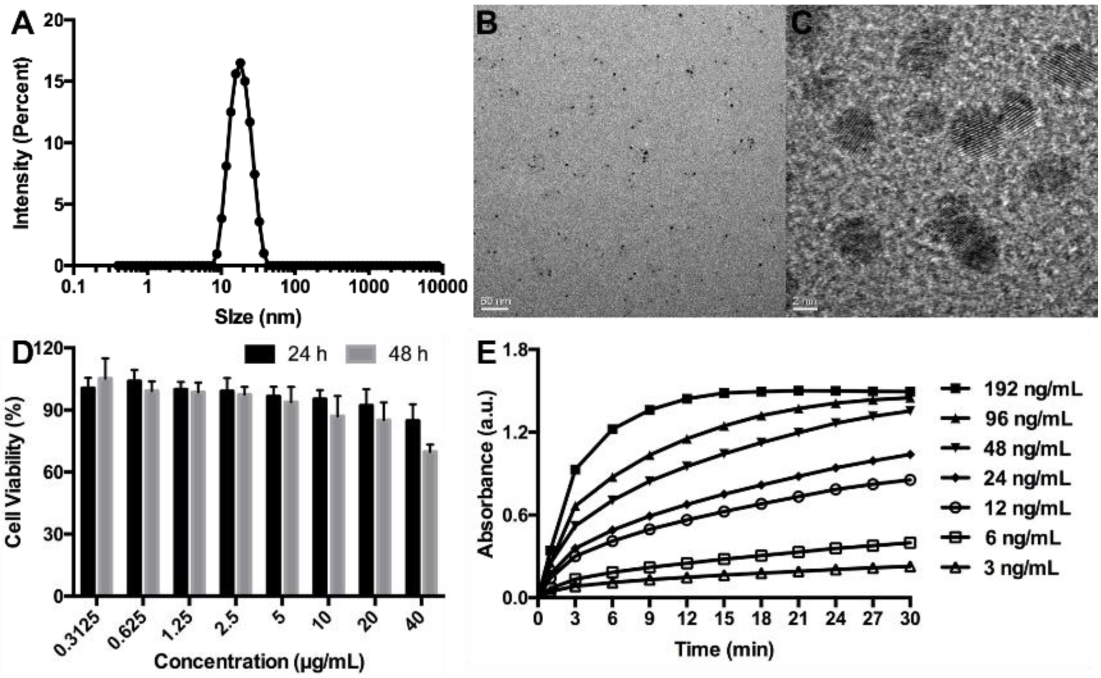

Figure S4. Characterization of PtNP. (A) Particle size, (B, C) TEM image, (D) *in vitro* cell viability of PtNP, and (E) the absorbance evolution for different concentrations of PtNP at 652 nm. Data in (D) and (E) are presented as mean ± SD (n = 6).

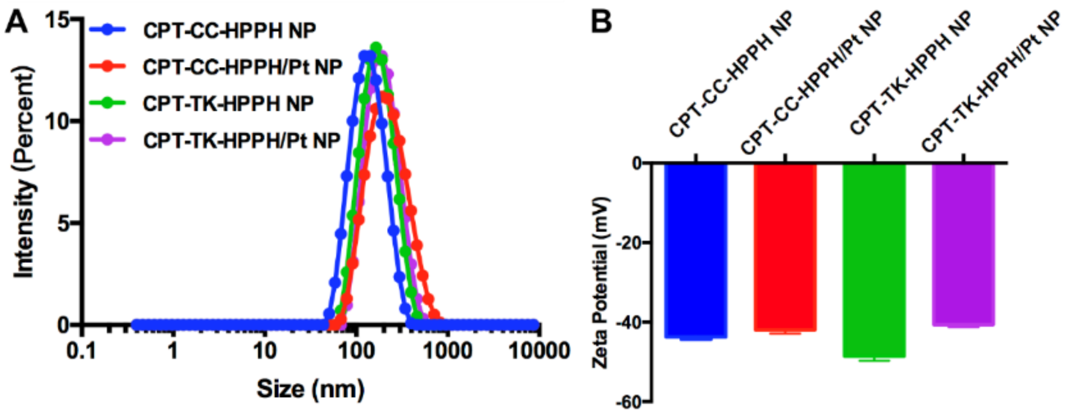

Figure S5. (A) Particle size and (B) zeta potential of CPT-CC-HPPH NP, CPT-CC-HPPH/Pt NP, CPT-TK-HPPH NP, and CPT-TK-HPPH/Pt NP. All quantitative data are presented as mean ± SD (n = 3).

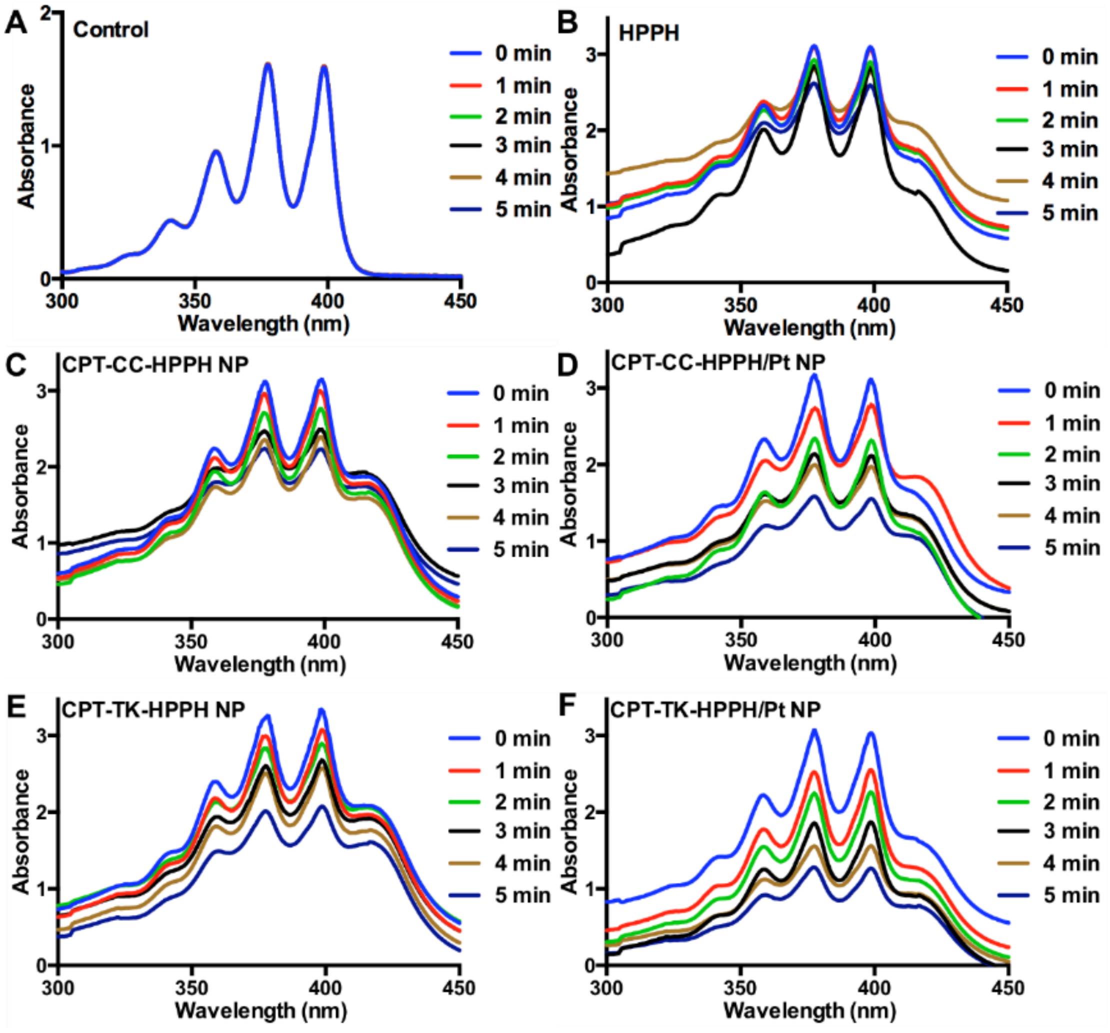

Figure S6. The UV-vis absorption spectra of ABDA in (A) control, (B) HPPH, (C) CPT-CC-HPPH NP, (D) CPT-CC-HPPH/Pt NP, (E) CPT-TK-HPPH NP, and (F) CPT-TK-HPPH/Pt NP group with 100 μM H_2O_2 at designated time irradiation for 660 nm laser.

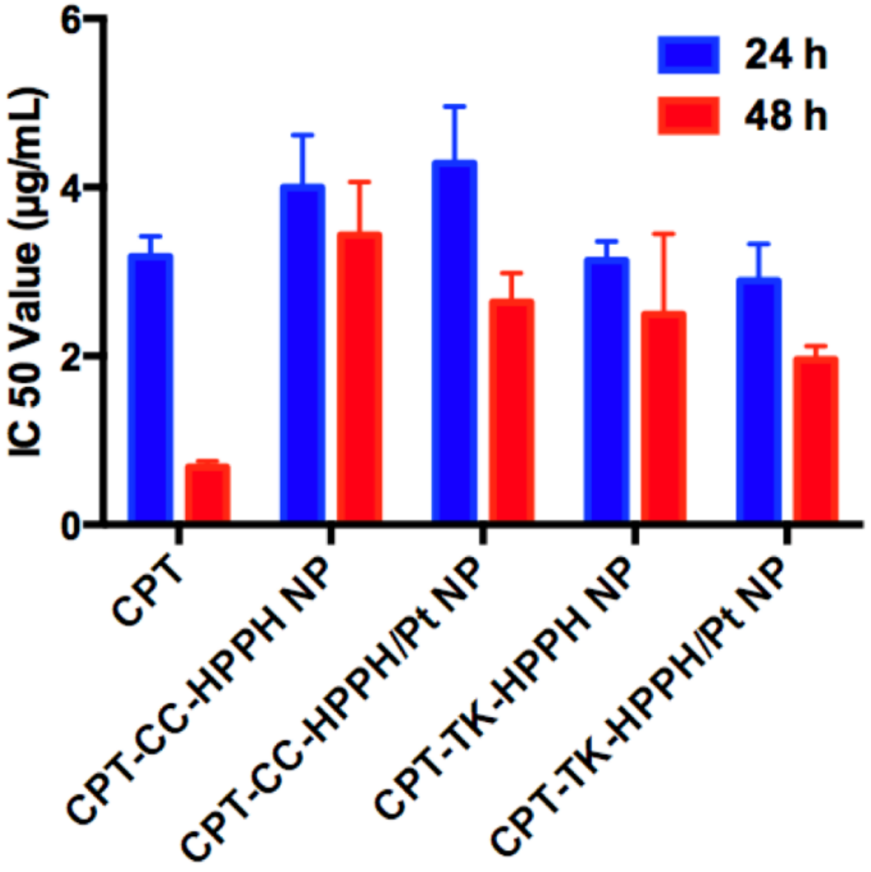

Figure S7. IC50 value of CPT, CPT-CC-HPPH NP, CPT-CC-HPPH/Pt NP, CPT-TK-HPPH NP, and CPT-TK-HPPH/Pt NP. The data are presented as mean ± SD (n = 6).

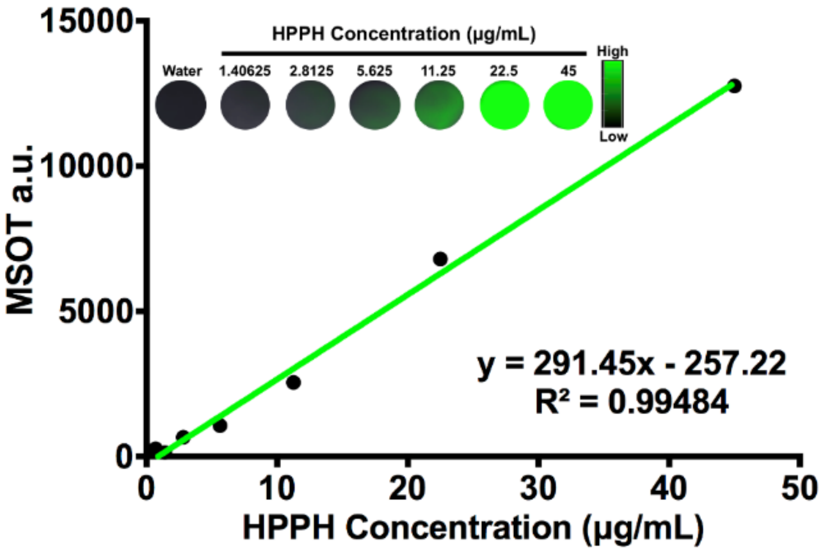

Figure S8. (A) Linear correlation of PA signals of HPPH in CPT-TK-HPPH/Pt NP against its corresponding concentrations. PA imaging of CPT-TK-HPPH/Pt NP at indicated HPPH concentrations at 680 nm, with the linear fitting equation inserted.

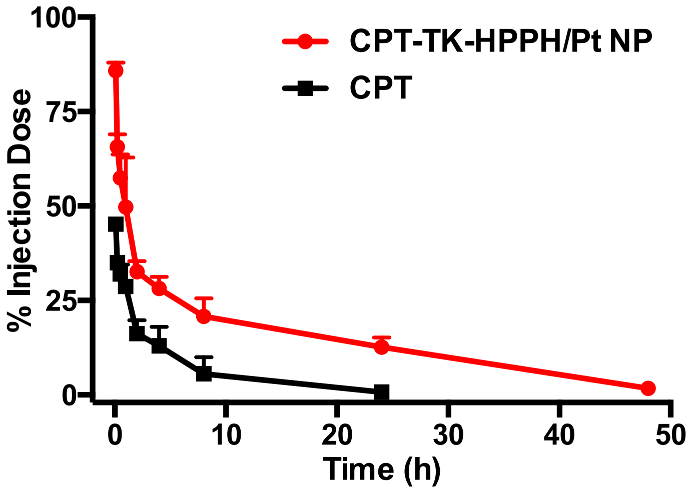

Figure S9. Plasma concentration-time profiles of CPT-TK-HPPH/Pt NP and CPT in rats after intravenous injection. The data are presented as mean ± SD (n = 3).

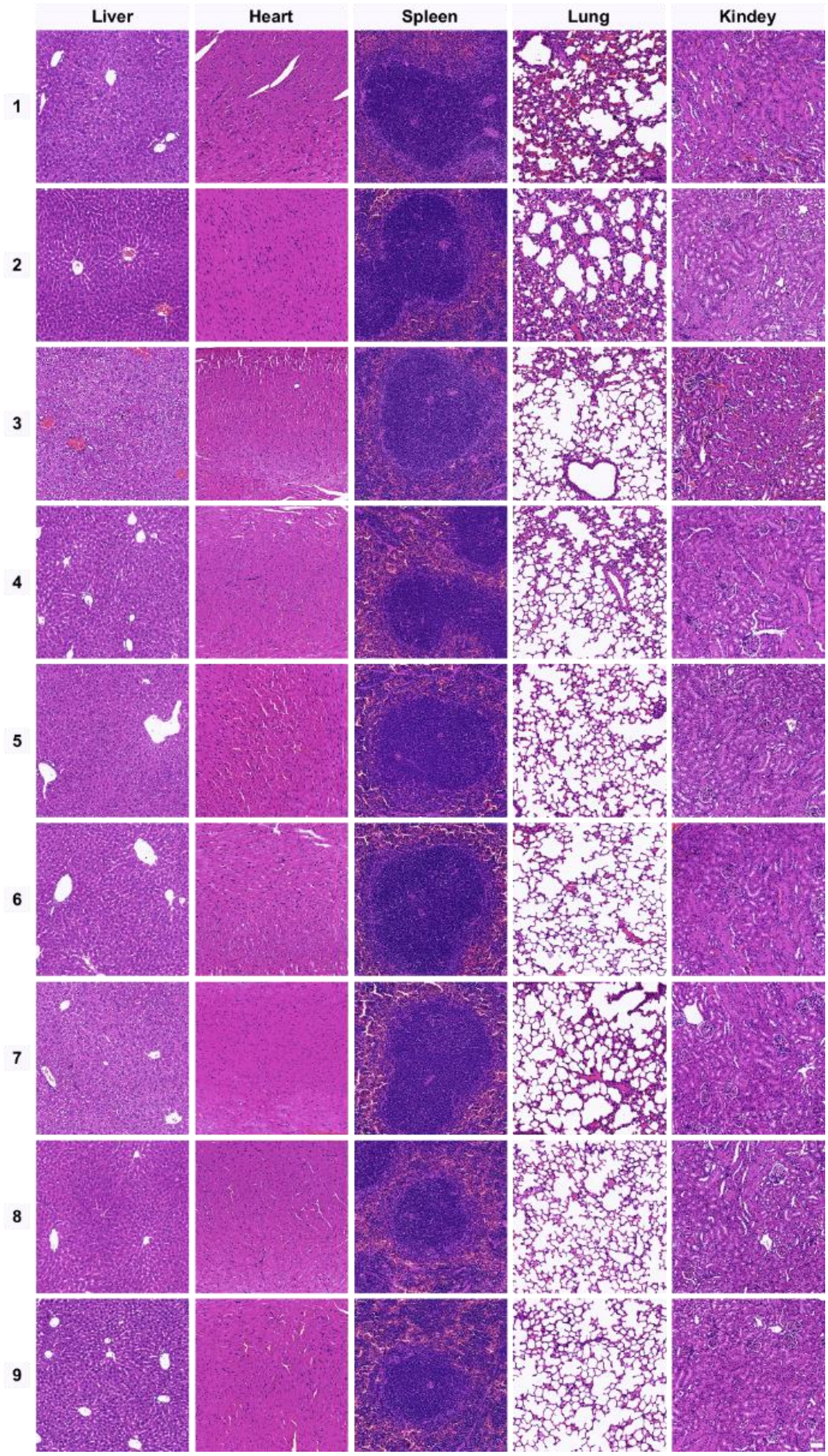

Figure S10. Representative H&E stained images of the liver, heart, spleen, lung, and kidney tissues (scale bar, 50 μm) of CT26 tumor-bearing mice. (1. Control, 2. Control+Laser, 3. CPT, 4. HPPH+Laser, 5. CPT-CC-HPPH/Pt NP+Laser, 6. CPT-TK-HPPH NP, 7. CPT-TK-HPPH NP+Laser, 8. CPT-TK-HPPH/Pt NP, 9. CPT-TK-HPPH/Pt NP+Laser.)

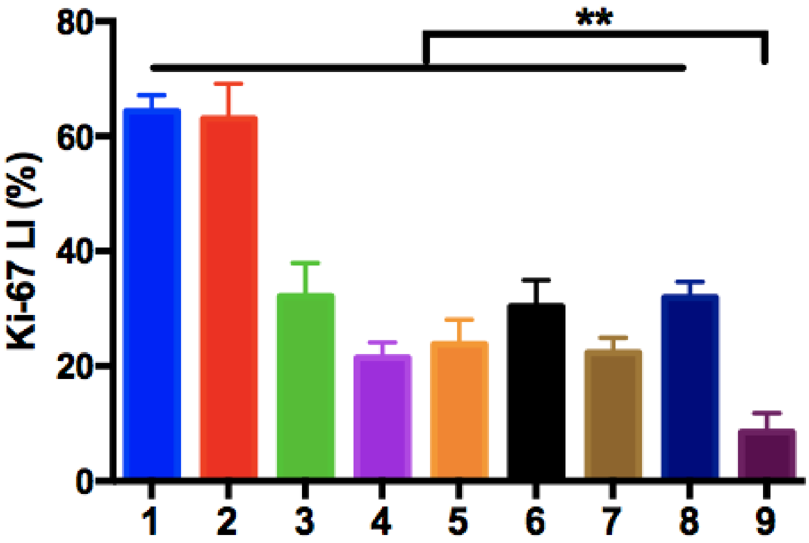

Figure S11. Ki-67 LI of tumors in each group. The data are presented as mean ± SD (n = 5). "**" means the P <0.01. (1. Control, 2. Control+Laser, 3. CPT, 4. HPPH+Laser, 5. CPT-CC-HPPH/Pt NP+Laser, 6. CPT-TK-HPPH NP, 7. CPT-TK-HPPH NP+Laser, 8. CPT-TK-HPPH/Pt NP, 9. CPT-TK-HPPH/Pt NP+Laser.)

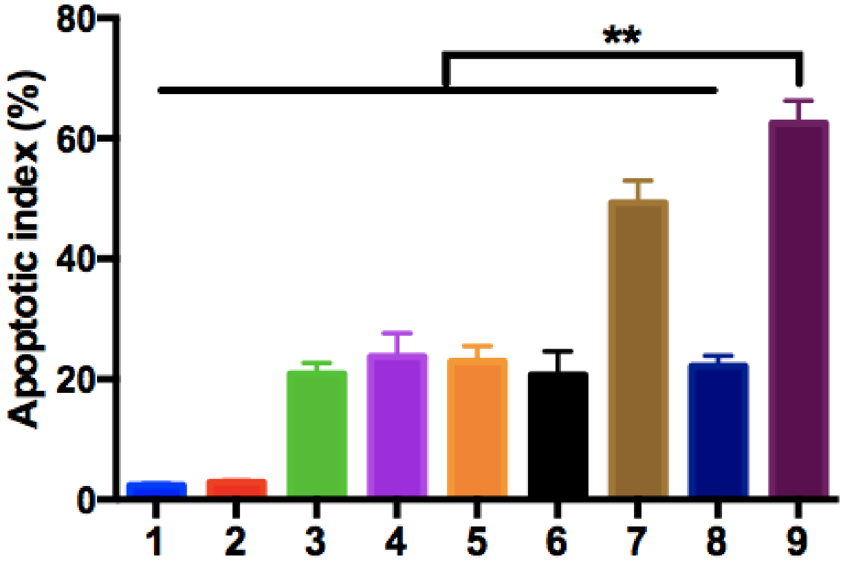

Figure S12. Apoptotic index of tumors in each group. The data are presented as mean ± SD (n = 5). "**" means the P <0.01. (1. Control, 2. Control+Laser, 3. CPT, 4. HPPH+Laser, 5. CPT-CC-HPPH/Pt NP+Laser, 6. CPT-TK-HPPH NP, 7. CPT-TK-HPPH NP+Laser, 8. CPT-TK-HPPH/Pt NP, 9. CPT-TK-HPPH/Pt NP+Laser.)
